# Three dimensional reconstruction of coronary artery stents from optical coherence tomography: *experimental validation and clinical feasibility*

**DOI:** 10.1038/s41598-021-91458-y

**Published:** 2021-06-10

**Authors:** Wei Wu, Behram Khan, Mohammadali Sharzehee, Shijia Zhao, Saurabhi Samant, Yusuke Watanabe, Yoshinobu Murasato, Timothy Mickley, Andrew Bicek, Richard Bliss, Thomas Valenzuela, Paul A. Iaizzo, Janaki Makadia, Anastasios Panagopoulos, Francesco Burzotta, Habib Samady, Emmanouil S. Brilakis, George D. Dangas, Yves Louvard, Goran Stankovic, Gabriele Dubini, Francesco Migliavacca, Ghassan S. Kassab, Elazer R. Edelman, Claudio Chiastra, Yiannis S. Chatzizisis

**Affiliations:** 1 Cardiovascular Biology and Biomechanics Laboratory, Cardiovascular Division, University of Nebraska Medical Center, 982265 Nebraska Medical Center, Omaha, NE 68198 USA; 2 Department of Cardiology, Teikyo University Hospital, Tokyo, Japan; 3 Department of Cardiology, National Hospital Organization Kyushu Medical Center, Fukuoka, Japan; 4 Boston Scientific Inc, Maple Grove, MN USA; 5 Medtronic Inc, Santa Rosa, CA USA; 6 Visible Heart Laboratory, Department of Biomedical Engineering, University of Minnesota, Minneapolis, MN USA; 7 Department of Cardiovascular Sciences, Fondazione Policlinico Universitario A. Gemelli IRCCS Università Cattolica del Sacro Cuore, Rome, Italy; 8 School of Medicine, Emory University, Atlanta, GA USA; 9 Minneapolis Heart Institute, Minneapolis, MN USA; 10 Department of Cardiovascular Medicine, Mount Sinai Hospital, New York City, NY USA; 11 Institut Cardiovasculaire Paris Sud, Massy, France; 12 Department of Cardiology, Clinical Center of Serbia, Belgrade, Serbia; 13 Laboratory of Biological Structure Mechanics (LaBS), Department of Chemistry, Materials and Chemical Engineering “Giulio Natta,”, Politecnico di Milano, Milan, Italy; 14 California Medical Innovation Institute, San Diego, CA USA; 15 Institute for Medical Engineering and Science, Massachusetts Institute of Technology, Boston, MA USA; 16 PoliToBIOMed Lab, Department of Mechanical and Aerospace Engineering, Politecnico di Torino, Turin, Italy

**Keywords:** Interventional cardiology, Biomedical engineering

## Abstract

The structural morphology of coronary stents (e.g. stent expansion, lumen scaffolding, strut apposition, tissue protrusion, side branch jailing, strut fracture), and the local hemodynamic environment after stent deployment are key determinants of procedural success and subsequent clinical outcomes. High-resolution intracoronary imaging has the potential to enable the geometrically accurate three-dimensional (3D) reconstruction of coronary stents. The aim of this work was to present a novel algorithm for 3D stent reconstruction of coronary artery stents based on optical coherence tomography (OCT) and angiography, and test experimentally its accuracy, reproducibility, clinical feasibility, and ability to perform computational fluid dynamics (CFD) studies. Our method has the following steps: 3D lumen reconstruction based on OCT and angiography, stent strut segmentation in OCT images, packaging, rotation and straightening of the segmented struts, planar unrolling of the segmented struts, planar stent wireframe reconstruction, rolling back of the planar stent wireframe to the 3D reconstructed lumen, and final stent volume reconstruction. We tested the accuracy and reproducibility of our method in stented patient-specific silicone models using micro-computed tomography (μCT) and stereoscopy as references. The clinical feasibility and CFD studies were performed in clinically stented coronary bifurcations. The experimental and clinical studies showed that our algorithm (1) can reproduce the complex spatial stent configuration with high precision and reproducibility, (2) is feasible in 3D reconstructing stents deployed in bifurcations, and (3) enables CFD studies to assess the local hemodynamic environment within the stent. Notably, the high accuracy of our algorithm was consistent across different stent designs and diameters. Our method coupled with patient-specific CFD studies can lay the ground for optimization of stenting procedures, patient-specific computational stenting simulations, and research and development of new stent scaffolds and stenting techniques.

## Introduction

Coronary artery stents have revolutionized the field of interventional cardiology. The structural morphology of coronary stents (e.g. stent expansion, lumen scaffolding, strut apposition, tissue protrusion, side branch jailing, strut fracture), and the local hemodynamic environment after stent deployment are key determinants of procedural success and subsequent clinical outcomes^1–7^. High resolution intracoronary imaging with optical coherence tomography (OCT) and high definition intravascular ultrasound (IVUS) is indispensable in stenting optimization^8^. The cross-sectional imaging of deployed stents by OCT or IVUS provides important information on stent morphology. However, it fails to elucidate the global spatial distribution of the stent in relation to the lumen and side branches, and does not provide any information regarding the local hemodynamic environment within the stent (macroenvironment) and around the stent struts (microenvironment). Even though the commercial OCT console provides 3D rendering of the stent and lumen in a straight line, the true 3D configuration of the stent and lumen cannot be appreciated with this method and the operator has no access to raw stent geometrical data. Therefore, further analysis or computational fluid dynamics (CFD) is impossible. A platform for precise three-dimensional (3D) reconstruction of coronary stents, combined with 3D reconstruction of the arterial lumen, may unlock a wide spectrum of information and lay the ground for optimization of stenting procedures, patient-specific computational stenting simulations, and research and development of new stent scaffolds and stenting techniques. To the best of our knowledge, there have been very few attempts for OCT-based 3D reconstruction of coronary stents^9–13^. These studies had several limitations due to insufficient validation, lack of clinical feasibility, and use of older generation stent designs deployed in straight lumen vascular geometries.

The aim of this work was to build upon the current state-of-the-art and accomplish the following: (1) Present a novel algorithm for 3D stent reconstruction of coronary artery stents, (2) Experimentally test the accuracy (at strut level) and reproducibility of this algorithm in patient-specific silicone coronary artery models, (3) Test the feasibility of the algorithm in diseased coronary artery bifurcations from actual patients, and (4) Test the feasibility of performing CFD studies in stent models 3D reconstructed with our algorithm. Notably, in this study we used stents from different vendors to highlight the versatility of our method.

## Methods

All methods were carried out in accordance with the relevant guidelines and regulations. The OCT and angiography data used in the experimental and clinical studies of this work were obtained from the PROPOT trial (randomized trial of the proximal optimization technique in coronary bifurcation lesions). The study was approved by the ethics committee of Teikyo University (IRB approval number 15-159-2) and informed consent was obtained from all study participants.

### Experimental studies

#### Experimental coronary artery models, flow chamber studies and imaging procedures

**Figure 1:**
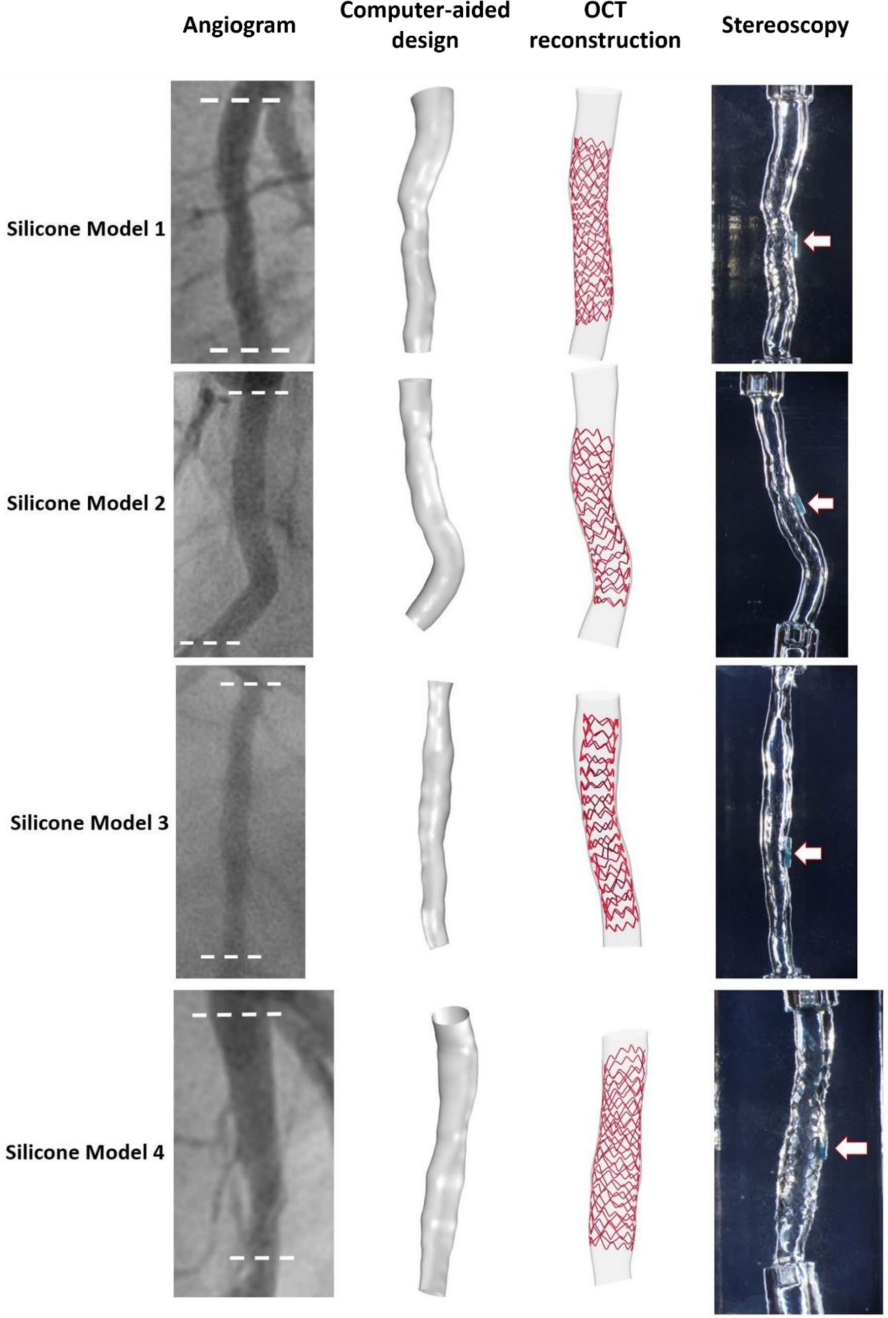
Patient-specific silicone models. In each model from left to right: angiography, computer-aided design before stenting, OCT-reconstructed lumen after stenting, and stereoscopy images of the final stented models. In stereoscopy images, the arrows indicate the markers used as reference for the orientation of the 3D reconstructed lumen.

We created four patient-specific silicone models of coronary artery lumen (Fig. 1), using our in-house technique as previously described^14^. We included both straight and curved coronary artery segments. In brief, the 3D lumen geometries were created in 3D CAAS Workstation 8.2 (Pie medical imaging, Maastricht, The Netherlands) using two different angiographic projections. Negative molds were designed according to the geometries and were 3D printed with acrylonitrile butadiene styrene. Polydimethylsiloxane was mixed with its curing agent and then poured into the dry clean molds. After curing, these silicone models were moved to an acetone beaker to dissolve the acrylonitrile butadiene styrene material. A small plastic marker was embedded in the silicone models to facilitate the correct orientation of the segmented OCT frames as described below.

The silicone models were placed in a custom-made flow chamber, and a bioreactor circuit was connected to the inlet and outlet of the flow chamber, allowing circulation at a steady flow rate of 100 ml/min at room temperature. Three different widely used second-generation stents (Synergy, Boston Scientific, Maple Grove, MN, USA; Resolute Onyx and Resolute Integrity, Medtronic, Santa Rosa, CA, USA) with diameters ranging from 2.5 mm to 4.0 mm were directly implanted into the silicone models (Table 1). In order to assess the ability of our method to reproduce any strut malapposition, post dilatations were not performed.

**Table 1:** Stent types and length measurement.

	Stent	Inflation pressure	OCT frame distance (mm)
Model 1	Resolute Integrity 3.5 × 18 mm	16 atm	0.1
Model 2	Synergy 3.0 × 16 mm	18 atm	0.1
Model 3	Synergy 2.5 × 16 mm	18 atm	0.2
Model 4	Resolute Onyx 4.0 × 18 mm	16 atm	0.1

The stented silicone models were imaged with angiography at two different projections with at least 30° difference in viewing angles. OCT imaging of the stented models was obtained using the OPTIS Integrated System (Abbott, Chicago, IL, USA). The OCT catheter (Dragonfly, Optis Imaging Catheter) was advanced through a 6F guiding catheter and pulled back (automatic triggering by saline without contrast) at a speed of 18 mm/s for models 1, 2, and 4, and at a speed of 36 mm/s for model 3. We selected two different pullback speeds to assess the performance of our algorithm under different OCT pullback modes.

#### 3D reconstruction of silicone lumen

The technique for semi-automatic 3D reconstruction of the silicone lumen was previously described^14^ and summarized in Table 2. Briefly, the lumen centerline was generated from two different angiographic views (3D CAAS Workstation 8.2; and VMTK, Orobix, Bergamo, Italy) and served as the backbone of the lumen reconstruction. The lumen contours from the segmented OCT images (EchoPlaque 4.0, INDEC Medical System, Los Altos, CA, USA) were aligned along the centerline and oriented using the aforementioned marker as a reference point. The aligned lumen contours were finally lofted to build the 3D lumen surface.

**Table 2:** Steps of the proposed method for 3D stent reconstruction.

1. 3D lumen reconstruction 1.1 Angiogram segmentation 1.2 Centerline generation 1.3 OCT segmentation 1.4 Packaging, rotation and lumen reconstruction	2. 2D stent reconstruction 2.1. Stent contour and strut point segmentation in OCT images 2.2. Packaging, rotation, straightening and planar unrolling of strut points and contours 2.3. Planar stent wireframe reconstruction
3. 3D stent reconstruction with lumen 3.1. Rolling back of the planar stent wireframe with the 3D reconstructed lumen as reference 3.2. Stent volume reconstruction	

#### OCT strut segmentation and planar stent unrolling

**Figure 2:**
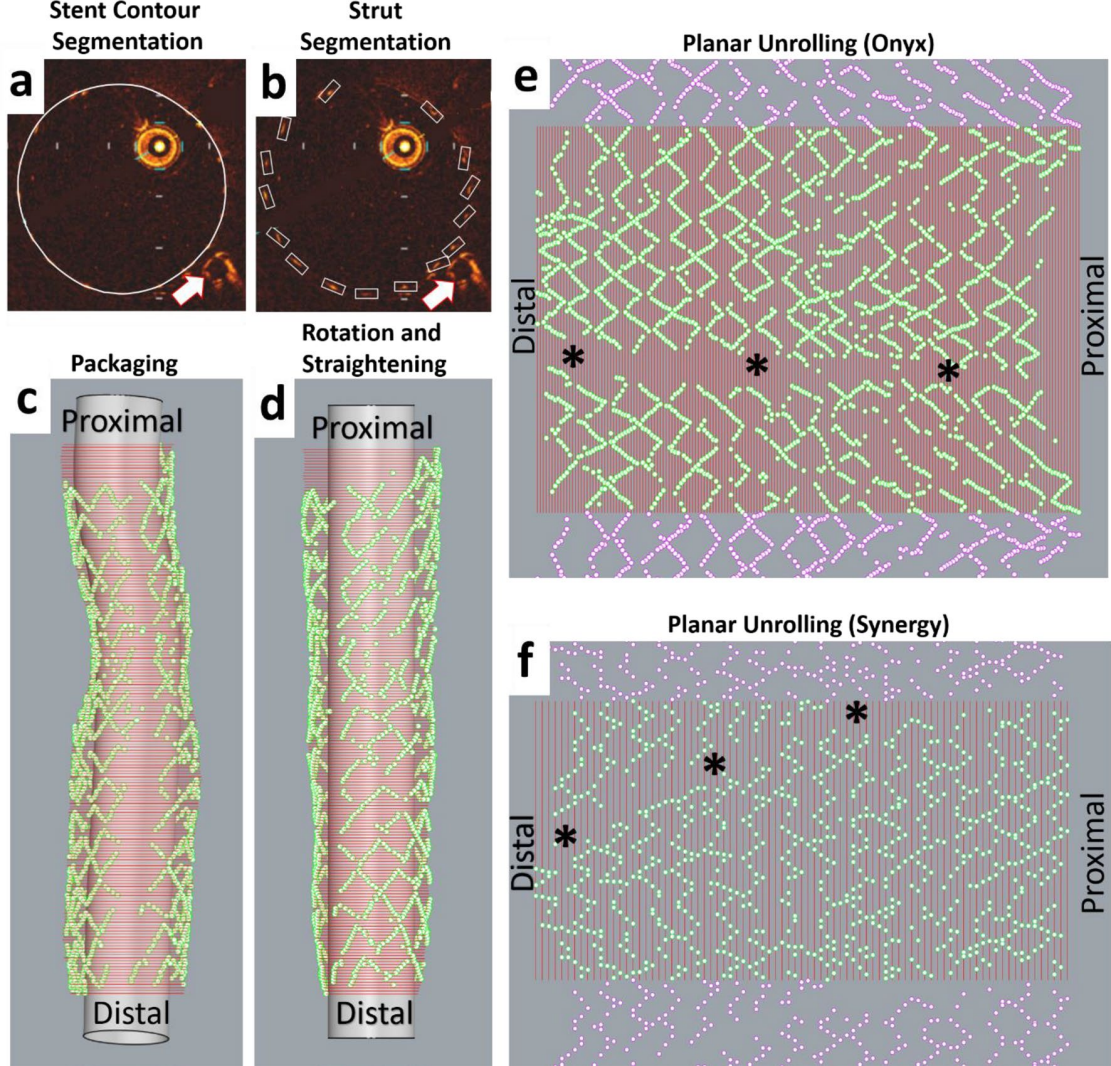
OCT stent segmentation and planar unrolling.** (a)** Segmented stent contour (white circle), and **(b)** segmented strut points (white boxes), and arrows indicate the markers used as reference for the orientation of the 3D reconstructed lumen, **(c)** Packaging of segmented stent contours and strut points in space, **(d)** Rotation and straightening of the packaged stent contours and strut points, **(e, f)** Planar unrolling (stent contours in red, stent strut points in green). The purple strut points were added to show the continuity of the stent structure. In **(e, f)**, note the zone of missing strut points corresponding to the wire shadow. **(a–e)** Depict Onyx stent and **(f)** Synergy stent.

All the steps were performed in Grasshopper 3D, a visual programming language and environment that runs within Rhinoceros 3D (Robert McNeel & Associates, Seattle, WA, USA). In each OCT frame used for the lumen reconstruction, we manually identified the stent contours and strut points (Fig. 2a,b). The segmented stent contours and strut points were imported into Grasshopper 3D. The segmented stent contours and strut points were packaged in a straight line along the OCT catheter center, rotated at the same angle with the corresponding lumen contour, and straightened, taking as reference the centroid of the distal stent contour (Fig. 2c,d). Finally, the stent contours and strut points were unrolled on a 2D plane (Fig. 2e,f).

#### Planar reconstruction of stent wireframe

**Figure 3:**
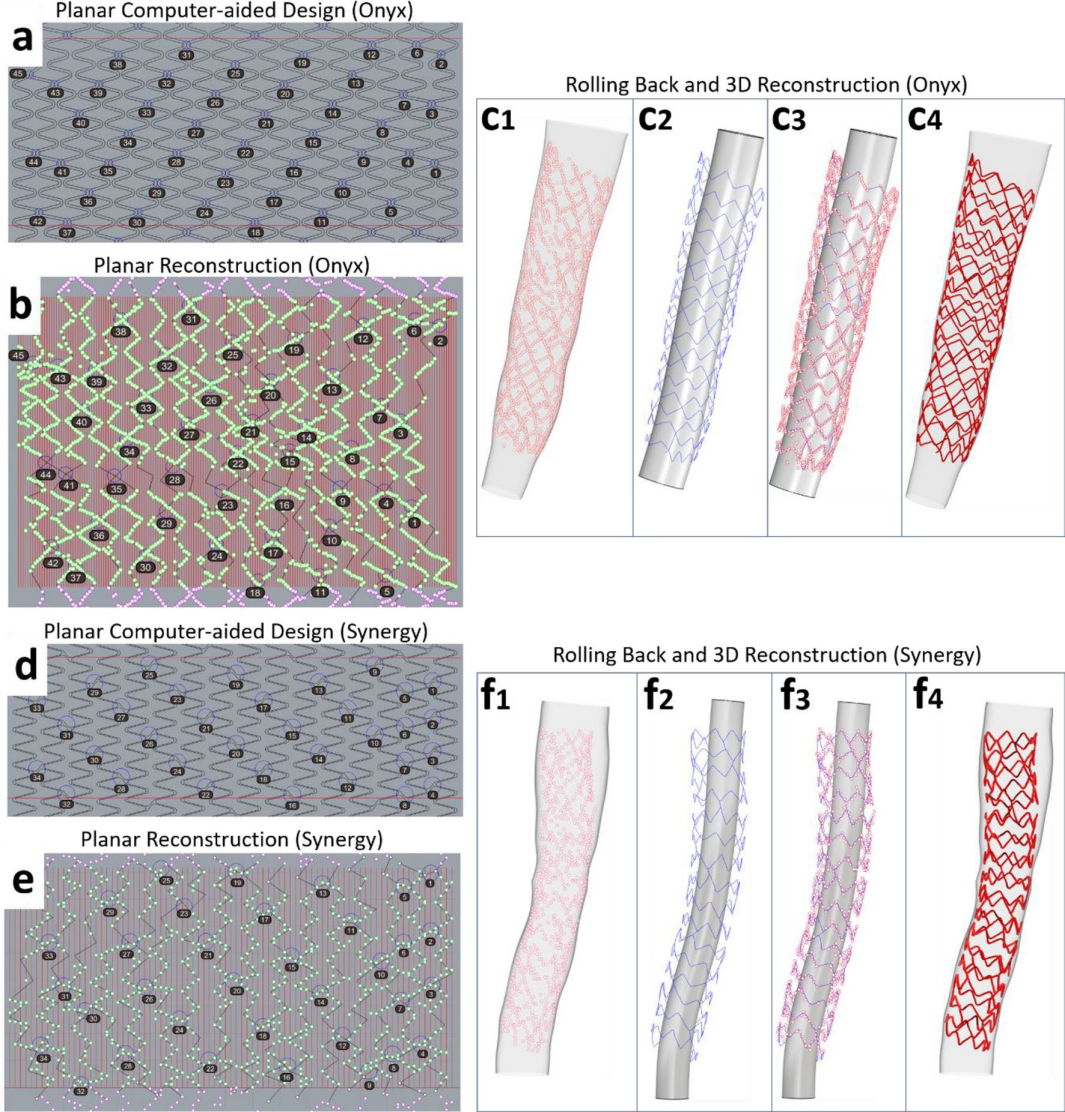
Planar stent wireframe reconstruction and rolling back to space. Planar computer-aided design of **(a)** Onyx stent and **(d)** Synergy stent with the boundaries delineated by red lines. Planar stent reconstruction (black lines) of **(b)** Onyx and **(e)** Synergy using the unrolled stent strut points and computer-aided stent designs as reference. The numbers and circles in **(a, b, d,** **e)** indicate the location of stent links. Note the extension of black lines beyond the stent boundaries to show the stent continuity for the next steps. Rolling back of 2D reconstructed **(c)** Onyx stent wireframe and **(f)** Synergy stent wireframe and volume creation: **(c1, f1)** 3D reconstructed lumen used as a backbone for the 3D stent reconstruction. The red points are segmented stent strut points, **(c2**, **f2)** 3D rolling-back of 2D reconstructed stent wireframe (blue) following the lumen shape, **(c3**, **f3)** Original strut points (red) overlapped on the rolled back stent wireframe, **(c4**, **f4)** Final stent volume generated from stent wireframe. A tube was added inside the stent wireframe for better visualization **(c2, c3, f2,** **f3**). Stent designs were provided by the stent manufacturers.

Following the planar flattening of stent struts, the 2D stent wireframe was reconstructed according to the corresponding planar computer-aided wireframe of the stent. The planar computer-aided designs of the stents were provided by the stent manufacturers (Fig. 3a,d). The stent links were marked with circles and numbered in an ascending order starting from the proximal or distal stent edge. The flattened strut points were connected with straight lines according to the following rules: (1) Each line fragment should connect consecutive “peaks” and “valleys” of the points, (2) The location and the sequence of the links (where the lines are connected) must be consistent with the planar computer-aided design of the stent, (3) In zones where strut points are not visible due to wire shadow, the operator should bridge the gaps by using as a reference the pattern of the “peaks” and “valleys” of the planar computer-aided designs of the stents, and iv) At the contour side boundaries, the lines must be repeated to maintain the stent structure continuity.

#### 3D stent reconstruction

The generated planar stent wireframe was rolled back in space and rounded at the peaks and valleys, using the 3D reconstructed lumen model as a backbone in Grasshopper 3D (Fig. 3c, f). The final stent volume was created by extruding the stent sections (circular for Onyx and Integrity, and rectangular for Synergy) along the 3D reconstructed stent geometry.

#### Micro-computed tomography (μCT) and stereoscopic imaging

The μCT and stereoscopic imaging of the stented silicone models were used as a reference for the validation of the stent reconstruction algorithm. μCT imaging (Skyscanner 1172 version 1.5) was performed with the following parameters: Image pixel size 26.94 μm, voltage 100 kV, current 100 μA, and slice thickness 27 µm. The stented models were 3D reconstructed from μCT images (Mimics 22.0, Materialise, Leuven, Belgium), and smoothened (Meshmixer, Autodesk Research, New York, NY, USA). Stereoscopic imaging was performed with an Olympus SZX16 camera (Tokyo, Japan) using a 6X magnification factor.

#### Experimental validation of the 3D stent reconstruction algorithm

The OCT-based 3D reconstructed stents were compared to their μCT reconstructed counterparts and to stereoscope imaging, using μCT and stereoscopy as references. For the quantitative comparison of OCT-based versus μCT-based reconstruction, the following metrics were used: (1) Stent length, (2) Mean stent diameter (MSD), defined as the average stent diameter of serial cross-sections every 0.1 mm along the stent length, (3) Stent shape, calculated by the ellipse ratio, i.e. the ratio of the maximum distance between the two furthest points of the stent cross-sectional circumference (distance X), and the maximum distance perpendicular to distance X (distance Y), and (4) Malapposition, calculated as the maximum distance between stent struts and lumen. To keep the analysis steps blinded, different operators performed the 3D reconstruction from OCT, 3D reconstruction from μCT, and comparison between OCT- and μCT-based models to minimize possible biases.

#### Reproducibility

To calculate the reproducibility of the OCT-based 3D reconstruction method, the stents were 3D reconstructed by two independent operators. The 3D reconstructed stents were compared in terms of MSD (quantitatively) and stent shape (qualitatively).

### Clinical Studies

#### Clinical Feasibility, Processing Times, and CFD Studies

The clinical feasibility and processing times of our stent reconstruction method were assessed in n = 3 patient coronary artery bifurcations with varying disease burden (Supplementary Table 1). All these cases underwent stenting with a single stent. OCT and angiography data were acquired according to the imaging protocols mentioned above. Both lumen and stent were 3D reconstructed following the steps of our proposed algorithm. To assess the time-efficiency of our stent reconstruction method, we calculated the processing time for each step in all three clinical cases.

Also, we assessed the feasibility of CFD studies in stents reconstructed with our method. The fluid domain was discretized into tetrahedral elements for the lumen (element size = 0.15 mm) and the stent (element size = 0.02 mm). Velocity inlet and outflow ratios were employed for the boundary conditions in which pulsatile flow of a human left coronary artery was used^15^, and the inlet velocity was tuned according to inlet diameter^16^. The outflow ratio was determined based on the diameter ratio of the left anterior descending and the left circumflex arteries^16^. Extensions of 10-diameter were added to the inlet and outlet sections to minimize the effect of boundary conditions. We considered blood as a Newtonian fluid with a density of 1050 kg/m^3^ and dynamic viscosity of 0.0035 Pa·s. The flow was considered to be laminar as the maximum Reynolds numbers of all cases at the maximum flow rate at the vessel inlet were less than the threshold of 2300. The simulations were performed for n = 3 cardiac cycles using a time step of 0.01 s, and only the last cycle results are shown. We calculated the time-averaged wall shear stress (TAWSS) using the following equation:

$TAWSS = \frac{1}{T}\mathop \smallint \limits_{0}^{T} \left| {\tau_{w} } \right|dt$, $\tau_{w}$ is the wall shear stress, T is the cardiac time cycle.

### Statistical analyses

Statistical analyses were performed with the statistical package GraphPad Prism 8.0 (GraphPad Inc., San Diego, CA, USA). Continuous variables are expressed as mean ± standard error of mean. For the validation and reproducibility studies, we used Bland–Altman analysis.

## Results

### Experimental validation

#### Stent morphology

**Figure 4:**
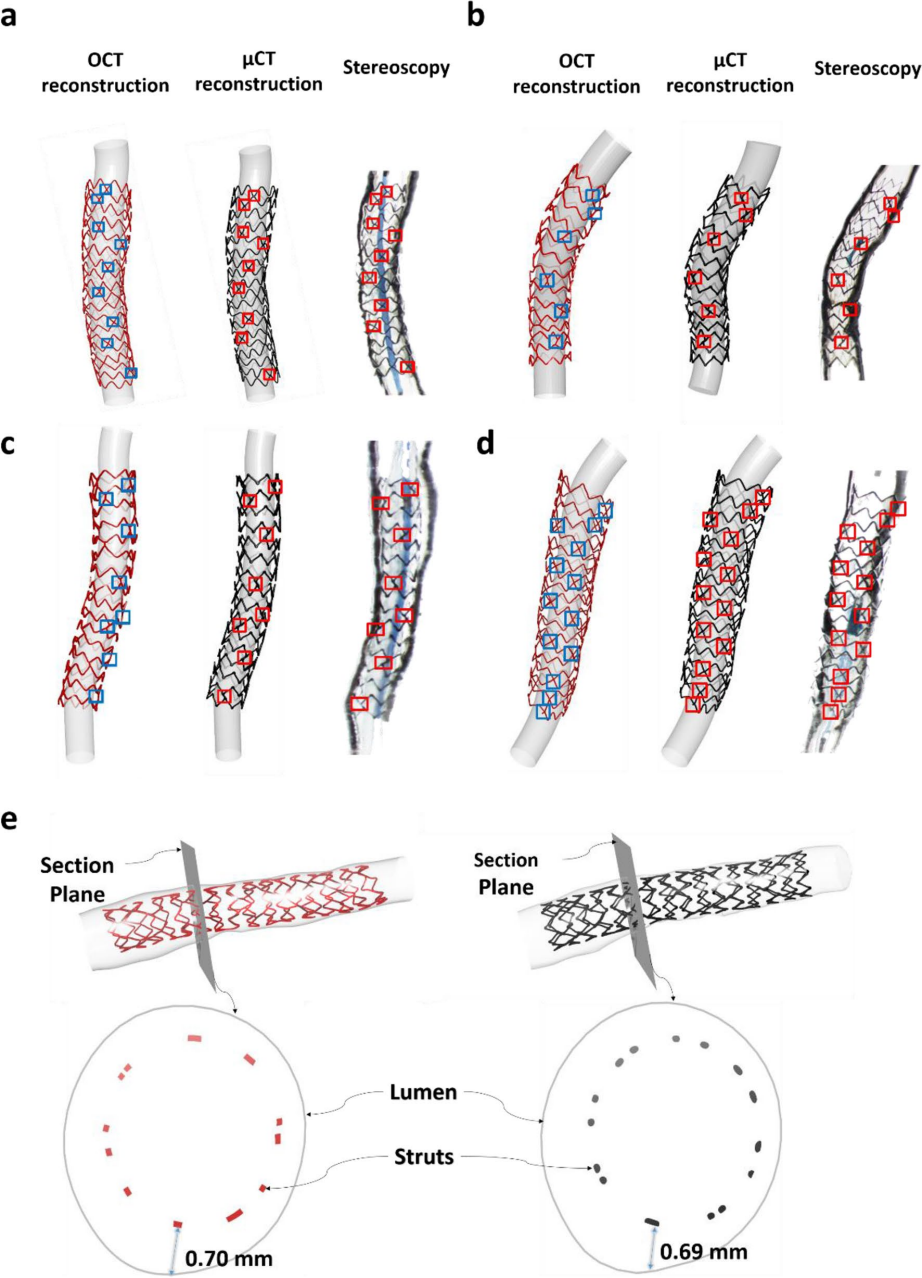
Qualitative experimental validation of stent reconstruction method against μCT and stereoscopic imaging.** (a–d)** Note the agreement in the link position (blue boxes in OCT-stent reconstructions and red boxes in μCT-stent reconstruction and stereoscopy). In OCT and μCT-based reconstructed models, a tube was inserted for better visualization, whereas in stereoscopy images, a small balloon was inflated in very low pressures for better visualization, **(e)** Malapposed struts in OCT-stent reconstruction (red stent) at the same location and magnitude compared to μCT-stent reconstruction (black stent).

All experimental stent models (n = 4) were successfully 3D reconstructed and compared to the corresponding μCT-reconstructed models and stereoscopy imaging (Fig. 4a–d). As indicated by the boxes, there was good agreement in terms of the location of the stent links between OCT-reconstructed stents, μCT-reconstructed stents and stereoscopy. This highlights the robustness of our method to faithfully reproduce the actual stent geometry at strut level.

#### Stent size, shape and malapposition

**Figure 5:**
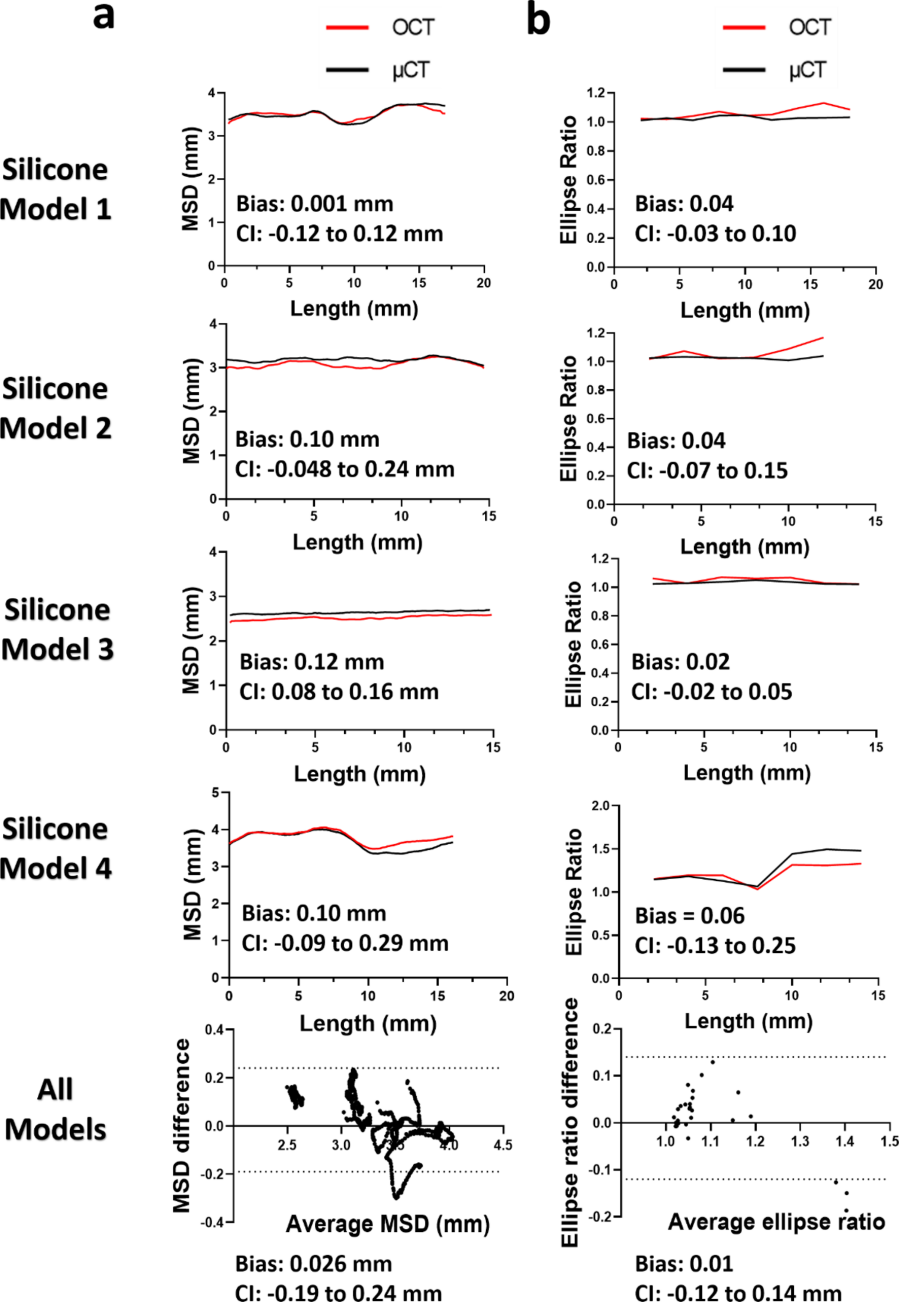
Quantitative experimental validation of stent reconstruction method against μCT. **(a)** Stent size measured by mean stent diameter (MSD), and **(b)** Stent shape measured by ellipse ratio.

There was high agreement in the stent length and MSD between the 3D reconstructed stents by OCT versus μCT (Table 3 for lengths, Fig. 5a for MSD). Bland–Altman analysis of the MSD between OCT- and µCT-reconstructed stents revealed a very small mean difference of 0.03 mm (95% CI from − 0.19 to + 0.24 mm). Similarly, the stent shapes were comparable to OCT and µCT reconstructions, yielding a mean difference of ellipse ratios of 0.01 (95% CI from − 0.12 to + 0.14 mm; Fig. 5b). Finally, our stent reconstruction method reproduced the severity of stent malapposition with high precision (Fig. 4e). These results strongly suggest the high accuracy of our method.

**Table 3:** Comparison of the length of the 3D reconstructed stents by OCT and μCT.

	Model 1	Model 2	Model 3	Model 4
Length by μCT (mm)	15.7	16.1	15.7	17.3
Length by OCT (mm)	15.8	15.7	15.8	17.3

#### Reproducibility

**Figure 6:**
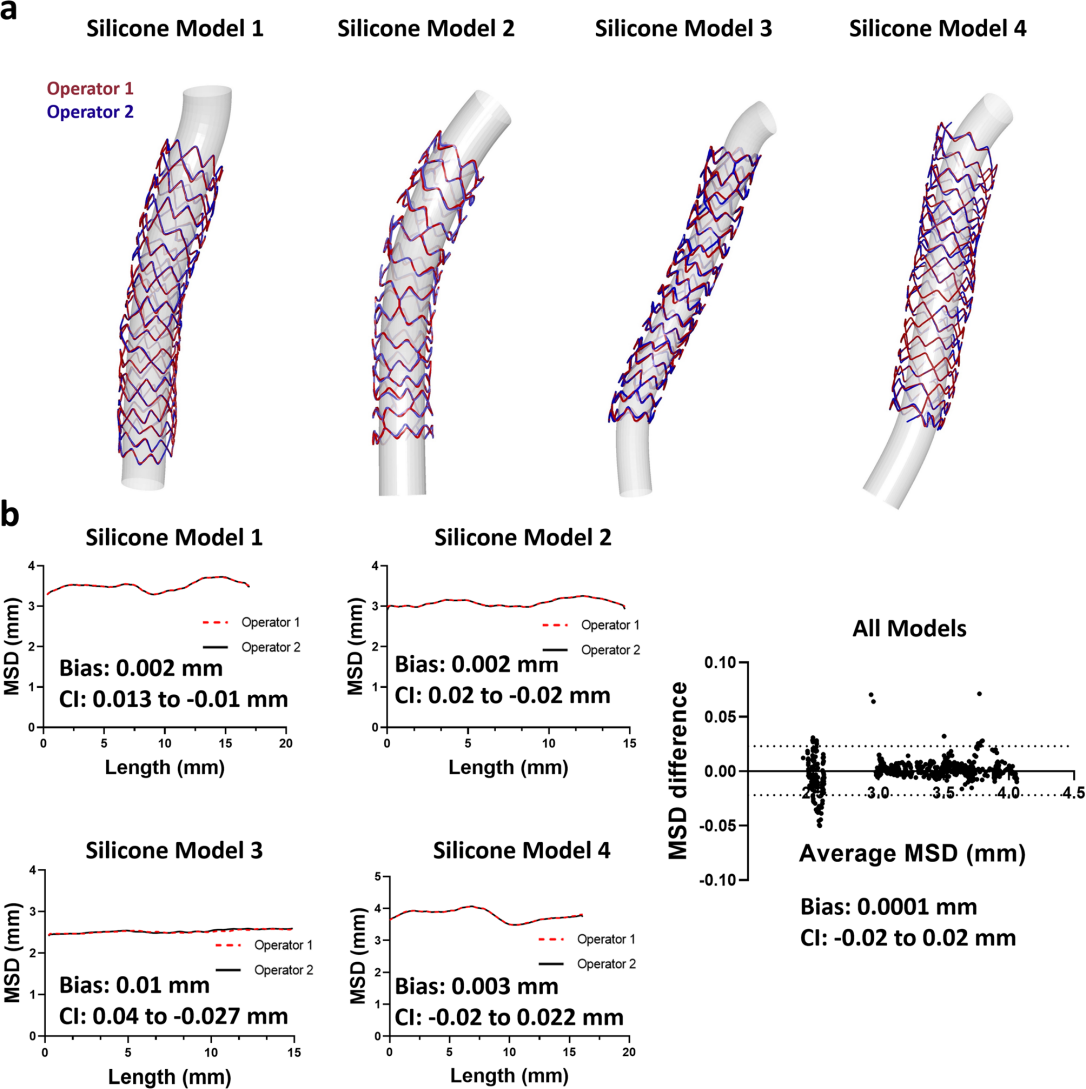
Inter-observer reproducibility of OCT-stent reconstructions. **(a)** Qualitative agreement by overlapping the two reconstructed stents. **(b)** Quantitative agreement for each model (MSD, mean stent diameter) and overall agreement using Bland–Altman analysis.

As shown in Fig. 6a, overlapping of the 3D reconstructed stents by two different operators yielded high interobserver reproducibility of our methodology. Quantitative comparison of MSD of the reconstructed stent models by two different operators showed that the graphs of MSD along the stent length were almost overlapping (Fig. 6b). Bland–Altman analysis revealed minimum mean differences in MSD of 0.0001 mm (95% CI from − 0.02 to + 0.02 mm), suggesting the very high reproducibility of the proposed stent reconstruction method.

### Clinical Feasibility and CFD Studies

**Figure 7:**
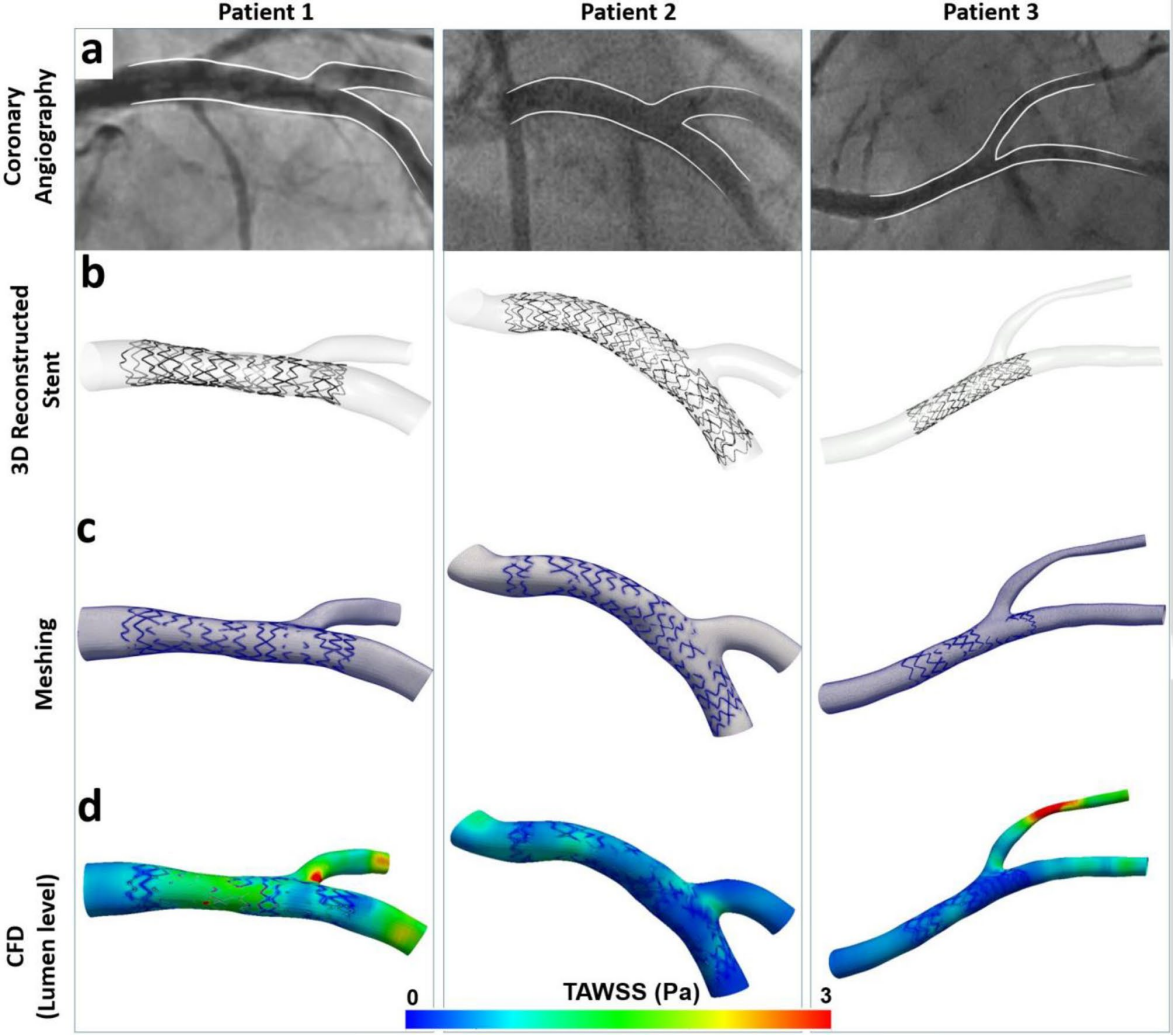
Clinical feasibility of the proposed 3D stent reconstruction method. **(a)** Invasive coronary angiography post stenting, **(b)** 3D lumen and stent reconstruction. Note the qualitative agreement to angiography, **(c)** meshing of a fluid domain in the stented lumen, **(d)** computational fluid dynamics (CFD) analysis depicting the time-averaged wall shear stress (TAWSS, Pa) along the stented bifurcations.

In all clinical cases (n = 3), the stents and corresponding coronary bifurcation lumens were successfully 3D reconstructed. The reconstructed stented bifurcations were qualitatively compared with the angiograms and showed good agreement in size and shape (Fig. 7a,b). The processing times for each step (from image processing to final 3D lumen and stent reconstruction) are summarized in Table 4. The average time for 3D reconstruction of stents was 177 ± 42 min, with longer stents requiring more processing time.

**Table 4:** Processing times for 3D stent reconstruction in clinical cases (n = 3).

Steps	Time (min)
**Step 1. 3D lumen reconstruction**	56 ± 5
**Step 2. 2D stent reconstruction**	
1. OCT stent segmentation	60 ± 10
2. Data importing and parameter setting	5 ± 1
3. Frame packaging, rotation, straightening and planar flattening	5 ± 1
4. Planar stent reconstruction	87 ± 18
**Step 3. 3D rolling back and stent volume creation**	20 ± 2
**Total time for 3D stent reconstruction (excluding step 1)**	177 ± 42
**Total time for whole process**	233 ± 20

**Figure 8:**
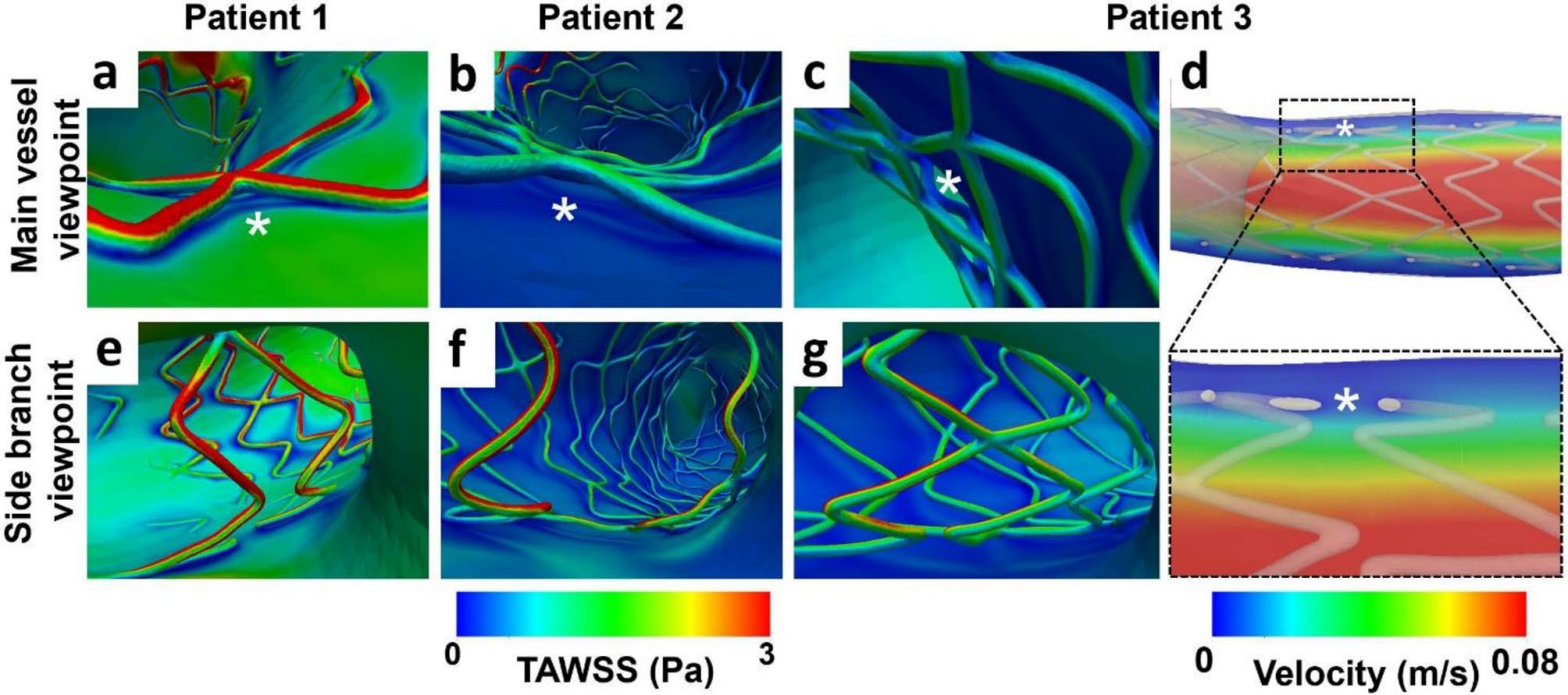
Representative examples of the calculated hemodynamic microenvironment (strut level) in patient-specific 3D reconstructed stented bifurcations.** (a–c)** The time-averaged wall shear stress (TAWSS, Pa) around stent struts. In **(a)** note the increased wall shear stress on the luminal surface of a well-apposed strut, and in **(b–c)** the wall shear stress distribution around malapposed struts denoted with white asterisks. In **(d)** longitudinal section showing the low velocity between the lumen and malapposed struts (white asterisks). **(e–g)** TAWSS distribution at the ostium of the side branch. Note the struts jailing the ostium of the side branch.

The reconstructed stented bifurcations were meshed (Fig. 7c) and underwent CFD studies. Figure 7d shows the TAWSS distribution along the macroenvironment of the 3D reconstructed stented bifurcation. Notably, disturbed flow was documented in the microenvironment of malapposed struts and the stented carina (Fig. 8).

## Discussion

In this study, we present a new methodology for the 3D reconstruction of coronary artery stents based on OCT and angiography. We assessed (i) The accuracy and reproducibility of the method using patient-specific silicone models of coronary arteries, and (ii) The clinical feasibility, time-efficiency, and ability to perform CFD studies using clinical bifurcation cases. For the validation studies, we used μCT and stereoscopy imaging as references. Our study showed that our algorithm: (1) Can reproduce the complex spatial stent configuration with high precision and reproducibility, (2) Is feasible in 3D reconstructing stents deployed in bifurcations, and (3) Enables CFD studies to assess the local hemodynamic environment at the lumen and strut level. Notably, the high accuracy of our algorithm was consistent across three contemporary second-generation stents (Synergy, Resolute Onyx, Resolute Integrity) with diameters ranging from 2.5 to 4.0 mm, supporting the versatility of our method.

To the best of our knowledge, this work overcomes several limitations of previous studies in the field and advances the current state-of-the-art. Table 5 provides a comprehensive head-to-head comparison of our study to the previous studies^10,12,13^ and the available software. The commercially available OCT console (OPTIS Integrated System, Abbott, Chicago, IL, USA) provides a real-time 3D reconstruction of the stent in a straight line. Using this software, the interventional cardiologist can easily assess multiple stent morphological parameters, including expansion, apposition, and side branch jailing at the point of care. However, the major disadvantage of the OCT console is that it cannot reconstruct the geometrically correct stent configuration; therefore, it cannot be used for CFD studies. Furthermore, this software is proprietary and the raw stent geometrical data are not accessible for further stent analysis and research. In contrast to the OCT console, our method reconstructs the true geometry of the stent that can be used for CFD studies. Of note, our algorithm is free and open to the scientific community. The 3D stent reconstruction methods proposed by Migliori et al.^12^ and Elliott et al.^10^ were based on geometrical information retrieved by OCT and μCT. Since μCT was an integral part of the reconstruction method, these techniques cannot be used in the clinical setting, and their applicability is limited to experimental research. However, a variation of the technique by Migliori et al.^12^ has been applied in clinical cases^9^. In contrast to these two studies, we used μCT solely for the purpose of validation in our method. Also, our method has direct and proven clinical applicability, given that it exclusively relies on angiography and OCT. Furthermore, Migliori et al. used older generation stent (Multi Link 8; Abbott Vascular), whereas in our study we used contemporary stents (Integrity, Onyx, Synergy) of varying size. The method proposed by O'Brien et al.^13^ is similar to our work in the use of OCT and angiography as source of anatomical information for 3D stent reconstruction. In fact, this work used an automatic technique for stent strut segmentation which is advantageous compared to our manual approach. However, the work by O'Brien et al.^13^ has some major limitations that we overcome in our study: O'Brien et al. work used outdated and relatively simple bare-metal stent designs, and its performance in current drug-eluting stent designs remains to be proven. The performance of O’Brien et al. strut mapping method in complex stent geometries and average quality OCT data appears questionable. On the contrary, our method performed well even with real-world average quality OCT data. Overall, the technically innovative approach of our method, coupled with the systematic validation and testing of its clinical feasibility using real-world, contemporary, second-generation stents, are notable advantages of our technique over the previous state-of-the-art.

**Table 5:** Comparison between different stent reconstruction methods (OCT: optical coherence tomography; μCT: micro-computed tomography; MSD: mean stent diameter; CFD: computational fluid dynamics; N/A: not available).

Study	Study data	Number of cases	Imaging modality	Segmentation technique	3D reconstruction technique	Processing time	Validation method	Clinical feasibility	Stent types tested	CFD analysis performed	Limitations
Wu et al (current study)	Bench and Clinical	7 (4 bench and 3 clinical)	OCT and Angiography	Manual segmentation (lumen, stent contour, stent struts)	2D manual framework + stent design; rolled back to 3D geometry	≈180 min	**Qualitative metrics** Final stent geometry vs. μCT, stereoscopy, angiography: Stent shape Stent links Strut apposition **Quantitative metrics** Final stent geometry vs. μCT: MSD Stent shape ratio Stent length	Yes	Synergy (Boston Scientific) Resolute Onyx (Medtronic) Resolute Integrity (Medtronic)	Yes	Long processing time
Elliott et al.^10^	Bench	4	OCT and μCT	Automatic segmentation (struts)	3D automatic mapping of known μCT derived stent design over the strut points	30 min	**Qualitative metrics** Pre-final geometry vs. segmented strut: Planar stent pattern **Quantitative metrics** Pre-final stent geometry vs. μCT derived stent wire-frame: Euclidean distance	No	Resolute Integrity (Medtronic) Xience Alpine (Abbott Vascular)	No	No clinical applicability since μCT is part of reconstruction method CFD analysis not performed Validation was not done for the 3D model
Migliori et al*.*^12^	Bench	1	OCT and μCT	Automatic segmentation (lumen, struts)	3D manual mapping of known stent design (in its straight expanded configuration) over the OCT strut points	N/A	**Qualitative metrics** Final geometry vs. μCT: Visual comparison **Quantitative metrics** Segmented strut point vs. μCT: Distance between the strut point and μCT	No	Multi Link 8 (Abbott Vascular)	Yes	Limited ability to reconstruct the stent in areas with shadow Old stent design used Long processing time
O’ Brien et al*.*^13^	Animal	4	OCT and Angiography	Automatic segmentation (lumen, struts)	3D automatic mapping of known stent design over the strut points	N/A	**Qualitative metrics** Final stent geometry vs. μCT: Visual comparison **Quantitative metrics** Final geometry vs. strut points: Displacement error between strut points and stent landmark Stent diameter Stent curvature	Yes	Small cell bare metal stent Large cell bare metal stent	Yes	Validated against automatic strut segmentation (Questionable ground truth) Old stents designs used
Commercial OCT console		N/A	OCT	Automatic segmentation (lumen, struts)	Not done	N/A	N/A	No	All commercially available stents	No	Straight stent reconstruction cannot be used for CFD Proprietary algorithm

Our methodology has several important technical aspects and innovations: First, it is based on a well-validated lumen reconstruction method^14^. The geometrically correct lumen reconstruction determines the location and orientation of the stent contours and struts points, and the relative location of the stent within the lumen. Second, our approach to unroll the stent contours and strut points and reconstruct the planar stent wireframe was a critical step that enabled faster and more accurate stent reconstruction. Direct 3D reconstruction based on the 3D strut segmentation appears to be technically challenging due to the geometrically complex distribution of strut points in space (Fig. 2). This becomes more pertinent at the gaps induced by the wire shadow and small red thrombus, where it is difficult for the operator to appreciate the correct 3D position of the missing strut points. However, when the strut points are unrolled on a 2D plane, it is much easier for the operator to find the stent pattern and fill the gaps, especially using the 2D stent design as reference (Fig. 3). Third, in our method, the stent links served as critical nodes for the precise planar reconstruction of the stent framework and subsequent 3D reconstruction of the stent (Fig. 3). Finally, all the steps in our method were automatic or semi-automatic except for the manual step to reconstruct the planar stent wireframe. Our code coordinated all these steps effectively, thereby reducing the human interaction and processing times and improving the accuracy and reproducibility of the proposed technique.

The proposed methodology has several scientific and clinical applications. It can be used at the cardiac catheterization laboratory to inform the interventional cardiologist on the spatial configuration of the deployed stent in relation to the main vessel lumen and side branches (when applicable), identifying areas of stent under-expansion, strut malapposition, and floating struts at the carina and side branch ostia. This information is essential for stenting optimization, which is directly related to favorable clinical outcomes^17,18^. Furthermore, our method can be combined with augmented reality techniques to allow for high-resolution visualization of the deployed stents for educational purposes. The proposed method can enable accurate CFD and solid mechanics studies to assess the local biomechanical environment after stenting, with emphasis on the strut level and the anatomically sensitive areas of bifurcations. Studies have associated areas with the disturbed flow and high shear rates with an increased propensity for stent restenosis and thrombosis^3,19^. Identification of these hemodynamically unfavorable areas within stents may enable stenting optimization and lead to improved clinical outcomes. Geometrically correct stent reconstructions can also provide important feedback to stent manufacturers regarding the design and performance of stents, enabling stent design optimization. Of note, our method has the potential to use other intracoronary imaging modalities, such as high definition IVUS, as anatomical input. The relatively simple steps of our method can allow operators without technical or engineering background (e.g., medical students, fellows) to use it, making it widely applicable.

This study has several limitations. First, the processing times were not optimal. The most time-consuming steps in the process were the: (1) Manual strut segmentation of OCT images (average time 60 ± 10 min), (2) Manual planar reconstruction of the stent wireframe (average time 87 ± 18 min), and the data transfer between different software used for the angiography and OCT image processing (i.e., CAAS, EchoPlaque, VMTK). Our current efforts focus on the development of a fully automated strut segmentation algorithm requiring limited manual interventions, a smart algorithm for planar stent wireframe reconstruction that limits the manual intervention to the highly challenging cases, and a new Grasshopper code to streamline the cross-talk between different software. We believe that these improvements will make our algorithm significantly faster. Second, our method required good quality OCT images. OCT images with incomplete blood clearance and severe image artifacts would not allow the operator to identify the stent struts making the planar reconstruction of the stent wireframe quite challenging. Finally, our technique was tested in single stent techniques/scenarios. The ability of our algorithm to reconstruct two stents (overlapping, bifurcation) is subject to future work.

## Conclusion

In conclusion, in this work, we propose a new method that enables accurate, reproducible, time-efficient and clinically feasible 3D reconstruction of coronary artery stents. This method, coupled with CFD studies, can facilitate stenting optimization, training in stenting techniques, and stent research and development.

## Supplementary Information

Supplementary Information.

## Abbreviations

3D: Three-dimensional
CFD: Computational fluid dynamics
IVUS: Intravascular ultrasound
MSD: Mean stent diameter
OCT: Optical coherence tomography
μCT: Micro-computed tomography
