# Supplementary material for: Three dimensional reconstruction of coronary artery stents from optical coherence tomography: experimental validation and clinical feasibility
Source: Sci Rep. 2021 Jun 10;11:12252. doi: 10.1038/s41598-021-91458-y (PMC8192920; doi:10.1038/s41598-021-91458-y)
Supplement: Supplementary file 1 — Supplementary Information. [file 41598_2021_91458_MOESM1_ESM.docx]

**Three Dimensional Reconstruction of Coronary Artery Stents from Optical Coherence Tomography: *Experimental Validation and Clinical Feasibility***

Wei Wu, PhD^1*^; Behram Khan, MD^1*^; Mohammadali Sharzehee, PhD^1^; Shijia Zhao, PhD^1^; Saurabhi Samant, MBBS^1^; Yusuke Watanabe, MD^2^; Yoshinobu Murasato, MD, PhD^3^;

Timothy Mickley, BSc^4^; Andrew Bicek, PhD^4^; Richard Bliss, BSc^5^; Thomas Valenzuela, BSc^6^; Paul Iaizzo, PhD^6^; Janaki Makadia, MBBS^1^; Anastasios Panagopoulos, MD^1^;

Francesco Burzotta, MD^7^; Habib Samady, MD^8^; Emmanouil S. Brilakis, MD, PhD^9^; George D. Dangas, MD, PhD^10^; Yves Louvard, MD^11^; Goran Stankovic, MD^12^; Gabriele Dubini, PhD^13^; Francesco Migliavacca, PhD^13^; Ghassan S. Kassab, PhD^14^; Elazer R. Edelman, MD, PhD^15^; Claudio Chiastra, PhD^16^; Yiannis S. Chatzizisis, MD, PhD^1#^

^1^Cardiovasclar Biology and Biomechanics Laboratory, Cardiovascular Division, University of Nebraska Medical Center, Omaha, Nebraska, USA

^2^Department of Cardiology, Teikyo University Hospital, Tokyo, Japan

^3^Department of Cardiology, National Hospital Organization Kyushu Medical Center, Fukuoka, Japan

^4^Boston Scientific Inc, Maple Grove, Minnesota, USA

^5^Medtronic Inc, Santa Rosa, California, USA

^6^Visible Heart Laboratory, Department of Biomedical Engineering, University of Minnesota, Minneapolis, Minnesota, USA

^7^Department of Cardiovascular Sciences, Fondazione Policlinico Universitario A. Gemelli IRCCS Università Cattolica del Sacro Cuore, Rome, Italy

^8^School of Medicine, Emory University, Atlanta, Georgia, USA

^9^Minneapolis Heart Institute, Minneapolis, Minnesota, USA

^10^Department of Cardiovascular Medicine, Mount Sinai Hospital, New York City, New York, USA

^11^Institut Cardiovasculaire Paris Sud, Massy, France

^12^Department of Cardiology, Clinical Center of Serbia, Belgrade, Serbia

^13^Laboratory of Biological Structure Mechanics (LaBS), Department of Chemistry, Materials and Chemical Engineering “Giulio Natta,” Politecnico di Milano, Milan, Italy

^14^California Medical Innovation Institute, San Diego, California, USA.

^15^Institute for Medical Engineering and Science, Massachusetts Institute of Technology, Boston, Massachusetts, USA

^16^PoliTo^BIO^Med Lab, Department of Mechanical and Aerospace Engineering, Politecnico di Torino, Turin, Italy

**Corresponding Author:**

*Yiannis S. Chatzizisis, MD, PhD*

Cardiovascular Biology and Biomechanics Laboratory

Cardiovascular Division, University of Nebraska Medical Center

982265 Nebraska Medical Center, Omaha, NE, 68198 USA

Tel: (402) 559-5156

Fax: (402) 559-8355

E-mail: [ychatzizisis@icloud.com](mailto:ychatzizisis@icloud.com)

**Supplementary Tables**

**Supplementary Table 1.** Clinical bifurcation cases (LCX: left circumflex artery; OM: Obtuse marginal artery; LAD: left anterior descending artery)

|  | **Bifurcation** | **Stent** |
| --- | --- | --- |
| Patient 1 | LCX/OM | Resolute Integrity 3.0x15 mm |
| Patient 2 | LAD/Diagonal | Resolute Integrity 3.5x22 mm |
| Patient 3 | LCX/OM | Resolute Onyx 3.0x15 mm |
